# Effects on collagen orientation in the cornea after trephine injury

**Published:** 2009-02-18

**Authors:** Christina S. Kamma-Lorger, Sally Hayes, Craig Boote, Manfred Burghammer, Michael E. Boulton, Keith M. Meek

**Affiliations:** 1 Structural Biophysics Group, School of Optometry and Vision Sciences, Cardiff University, Cardiff, Wales, UK; 2 Cell and Molecular Biology Group, School of Optometry and Vision Sciences, Cardiff University, Cardiff, Wales, UK; 3 Central Building, European Synchrotron Radiation Facility, Grenoble, France

## Abstract

**Purpose:**

Structural changes are well known to occur in the cornea after injury. The aim of this study was to investigate collagen orientation changes in the cornea during a short-term wound healing process.

**Methods:**

Seven bovine corneas were injured using a penetrating 5 mm biopsy punch and were subsequently organ cultured for up to two weeks. Six uninjured corneas acted as controls. The trephine wounded samples were snap frozen in liquid nitrogen either immediately after injury (0 h) or after 1 or 2 weeks in culture. Control/uninjured samples were snap frozen on arrival (0 h) or after 1 or 2 weeks in culture. Wide angle X-ray diffraction data were collected from each cornea at the UK Synchrotron Radiation Source or at the European Synchrotron Radiation Facility. Data analysis revealed information about collagen orientation and distribution in the corneal stroma during wound healing. For histology, two trephine wounded corneas at 0 h and 1 week and one control/uninjured cornea at 0 h were fixed in 10% neutral buffered formalin and processed for wax embedding. Wax sections were subsequently counterstained with haematoxylin and eosin to observe tissue morphology and the time course of complete re-epithelialization.

**Results:**

Immediately after injury, collagen organization was altered in a small area inside the wound but remained similar to the control/uninjured sample in the remainder of the tissue. After one week, the trephine wounded corneas showed complete re-epithelialization and evidence of swelling while collagen adopted a radial arrangement inside and outside the wound.

**Conclusions:**

Remarkable changes in collagen fibril orientation were observed in trephine wounded corneas. Orientation changes immediately after wounding are likely to be due to the mechanical deformation of the tissue during the wounding process. However, tissue swelling and changes in collagen orientation at later stages probably reflect the processes of tissue repair. These differences will determine corneal stability and strength following trauma and possibly refractive surgery.

## Introduction

The cornea and the sclera form the outer anterior surface of the eye. The thickest layer of the cornea is the stroma that consists mainly of water, collagen, proteoglycans, and keratocytes [1]. The precise architecture of the corneal stroma gives the tissue the appropriate curvature and transparency, essential for light focusing and normal visual function [2]. The human cornea consists of approximately 200 stacked lamellae that lie in parallel planes to the corneal surface [3]. Additionally, X-ray scattering techniques have revealed that collagen in central regions of healthy human corneas has a preferred orientation toward the superior–inferior and nasal–temporal directions [2,4,5]. The factors that affect collagen orientation during the wound healing process are still unknown, however, in vivo studies in rabbit corneas have shown that full penetrating wounds lead to changes in collagen orientation [6]. Given that the precise arrangement of stromal collagen is believed to largely govern corneal biomechanics, understanding collagen reorganization after wounding is an important step toward minimizing corneal biomechanical instability and changes in curvature following corneal surgery.

Wound healing is a complex process that demands to be performed in such a way that the clarity, strength, and stability of the tissue are not compromised in the long-term, especially in the ocular surface. In the cornea, the nature of the wound healing response appears to depend on the type and severity of injury [7-9]. Trephine wounding is a particularly severe method in which a biopsy punch is used to induce injury and the whole disc of tissue is excised from the sample.

In the current study, wide angle X-ray diffraction was used to obtain information about the predominant orientation of collagen within the cornea following trephine wounding and healing in a culture system, which was described previously by Foreman, et al. [10]. The aim of this work was to monitor the time course of collagen ultrastructural changes in cultured bovine corneas after being subjected to trephine injury.

## Methods

### Sample preparation and histology

Bovine eyes were obtained from a local abattoir and transported to the laboratory on ice. Thirteen healthy eyes with clear/transparent corneas were processed for organ culture as previously described [10] within 5 h of slaughter. A 5 mm surgical biopsy punch (Stiefel, High Wycombe, UK) was used to induce a circular wound in the middle of the cornea that extended approximately half-way through the stromal thickness. The 5 mm disc was removed using a scalpel blade. The eye was then dissected. The cornea with a scleral rim was excised and left to heal in culture. Seven trephine wounded corneal samples were wrapped in Clingfilm® (Superdrug, Croydon, UK), and snap frozen in liquid N_2_ immediately after injury (defined as 0 h; n=3) or after 1 week (n=3) or 2 weeks (n=1) in culture. Five unwounded, clear, control corneas were also dissected and snap frozen in liquid N_2_ immediately upon receipt from the abattoir (defined as 0 h) and another single cornea was snap frozen in the same manner following one week in culture. Samples were then kept at −80 °C until required for X-ray data collection. Unpublished studies conducted within our laboratory have shown that freezing and subsequent thawing does not affect the orientation of collagen. For histology, control and wounded samples were removed from organ culture at 0 h and 1 week and fixed in 10% neutral buffered formalin (NBF) solution overnight and processed/embedded in paraffin wax the next day. Wax cross sections (7 μm) of the cornea extending from limbus to limbus including the wound (i.e., trephine) were obtained, wax-cleared, and dehydrated. The sections were subsequently counterstained with hematoxylin and eosin to observe tissue morphology.

### X-ray diffraction

**Figure 1:**
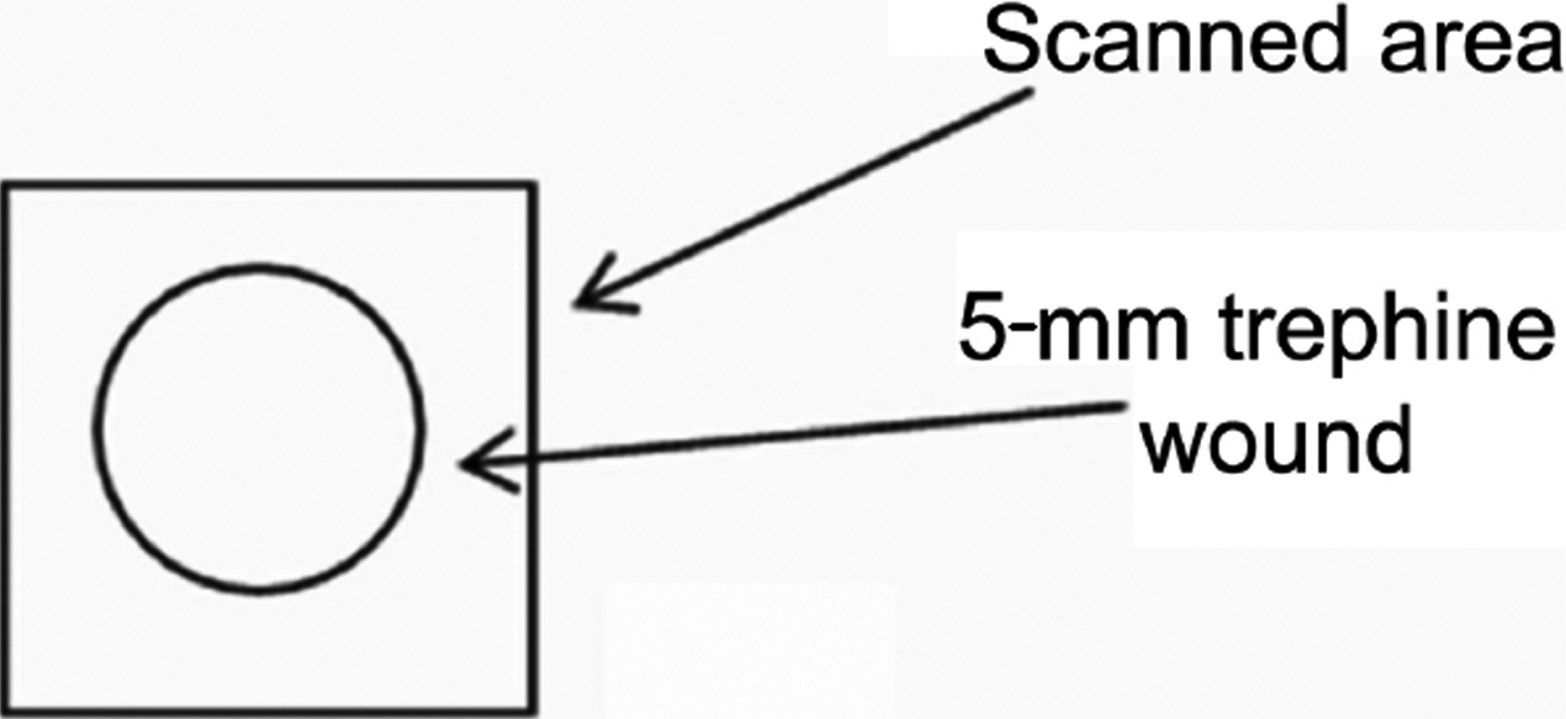
Square corneal piece from central cornea with the 5 mm trephine wound in the middle. The whole area of the corneal piece was scanned.

Wide angle X-ray diffraction data for each of the samples were collected either at station 14.1 (UK Synchrotron Radiation Source, Daresbury Laboratories, Warrington, UK) or at station ID13 in the European Synchrotron Radiation Facility (ESRF, Grenoble, France) using the method described by Aghamohammadzadeh et al. [5]. The bovine corneoscleral buttons had to be trimmed down to fit in the sample holder (Figure 1). For trephine wounded samples, a 6.9×6.9 mm^2^ or 7.2×7.2 mm^2^ corneal piece encompassing the wounded area was examined. During data collection, all samples were wrapped in Clingfilm^®^ and placed in airtight Perspex/Mylar chambers to prevent dehydration of the tissue. X-rays were passed through the anterior corneal face, parallel to the optical axis. For experiments conducted at the Daresbury SRS, a 1 mm^2^ cross-section X-ray beam (wavelength=0.1488 nm) was used and diffraction patterns recorded on a Quantum 4R CCD detector (ADSC, Poway, CA) situated 15 cm behind the cornea. Using an exposure time of 15-30 s data were collected at 0.3 mm intervals across 4 corneas and at 1 mm intervals across 5 other corneas. Preliminary experiments also involved the scanning of a 14 mm diameter central corneal area from a control/uninjured sample at 1 mm steps and an exposure time of 15 s. For experiments performed at the ESRF, an 8 μm^2^ beam (wavelength=0.1 nm) was used and diffraction patterns were recorded on a CCD FReLoN 2000 4M^™^ (Analog Transient Electronics Group, ESRF instrument support group, Grenoble, France) placed 18 cm behind the cornea. Data were collected at 0.15 mm (n=1), 0.3 mm (n=2), or 1 mm (n=1) steps across the tissue with an exposure time of 1 s. The specimen was translated in the vertical and horizontal directions within the corneal plane between exposures using a computerized translation stage interfaced with the camera shutter.

### Data analysis for wide angle X-ray diffraction

Analysis of X-ray data was performed using a Unix-based graphics program (Fit2D, produced by Dr Hamersley, ESRF) and Windows-based programs (Microsoft Excel [Reading, UK], Media Cybernetics Optimas 6.5 [Bethesda, MD], and StatSoft Statistica 6 [Bedford, UK]). To correct for beam current decay during data collection and variations in exposure time between samples, a normalization factor was calculated by multiplying the average beam current reading from each exposure by the exposure time in seconds (i.e., 1 s, 15 s, or 30 s).

**Figure 2:**
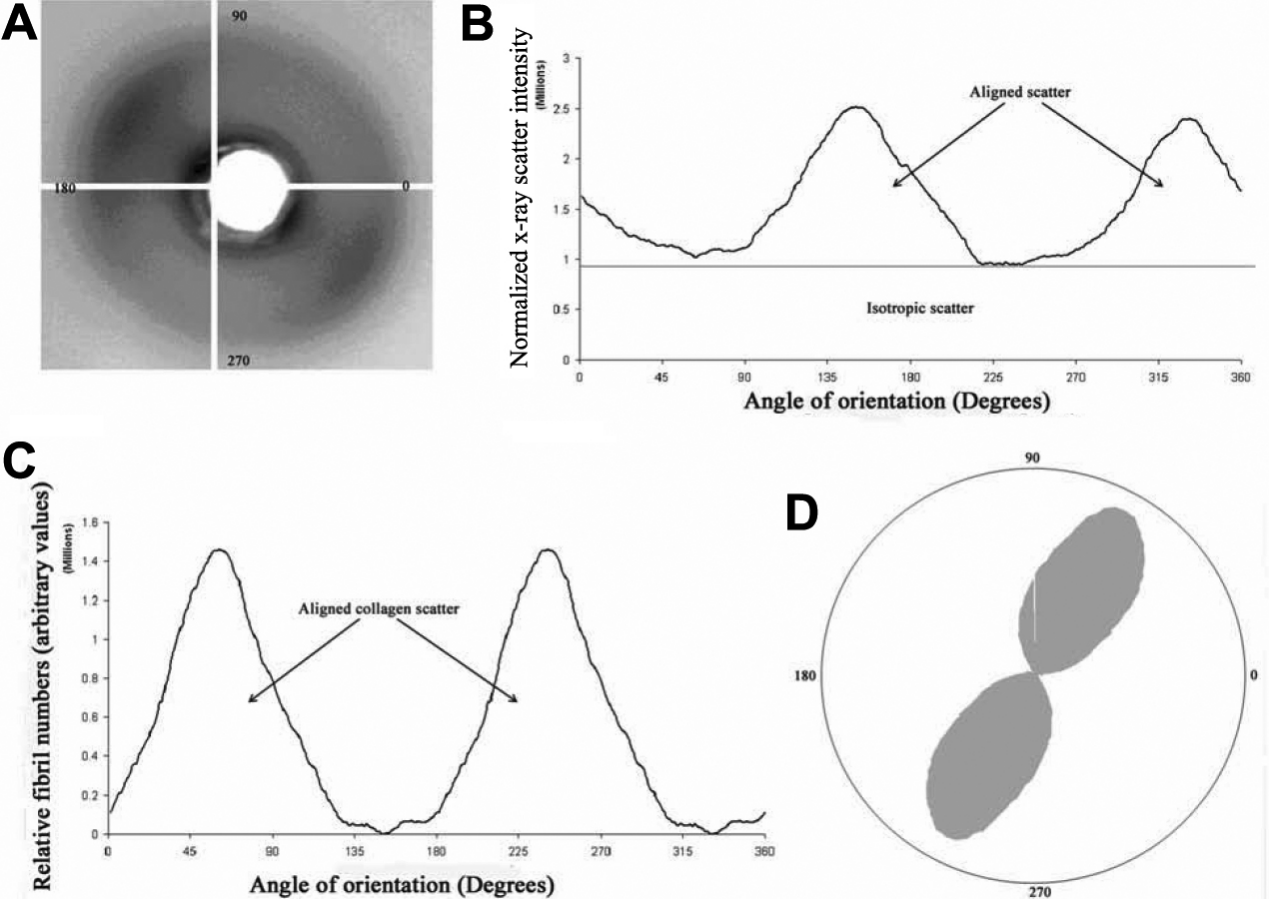
Diagram depicting the main stages of the wide angle X-ray diffraction analysis. An X-ray diffraction pattern (**A**) was obtained by passing the X-ray beam through the cornea parallel to the optical axis. The pattern was integrated radially across the width of the equatorial reflection and an intensity profile for each rotation angle between 0° and 360° was produced (**B**). The plot of integrated intensity as a function of rotation angle may be separated into two components, scatter from isotropically arranged collagen and scatter from preferentially aligned collagen [13] (Black horizontal line, **B**). After removal of the isotropic component, data were shifted by 90° to account for the fact that equatorial reflections occur at right angles to the collagen fibril axis [11]. The data (**C**) then represent the distribution of aligned collagen as a function of rotation angle, and this distribution may be expressed in polar coordinates [2] (**D**). The size of the polar plot in any given direction represents the amount of collagen preferentially oriented in that direction.

Corneal wide-angle diffraction patterns (Figure 2A) contain an equatorial X-ray reflection arising from X-rays scattered at right angles to the collagen molecules within fibrils. Each of the 200–400 lamellae in the path of the X-ray beam contributes to the equatorial diffraction pattern [11-13], and because lamellae occur at all angles within the plane of the cornea, this pattern takes a general circular form (Figure 2A). The scattered X-ray intensity was plotted as a function of radial distance from the image center for each pattern, and a radial background scatter function was fitted and subtracted, leaving only scatter from fibrillar collagen [14]. A single intensity value from each point of the circumference of the collagen reflection was obtained by integrating radially the background subtracted data. This was repeated for all rotation angles around the reflection, and a graph of collagen integrated X-ray scatter against angular position around the circular X-ray diffraction reflection was produced (Figure 2B). At this stage, the area below the graph is proportional to the total collagen mass [15] and comprises contributions from collagen lying in all directions (isotropic scatter) and fibrillar collagen with a preferred orientation (aligned scatter).

During the last stage of the analysis, polar plots were created to have a visual representation of the actual orientation distribution of collagen as well as its relative quantity in a given direction of preferred orientation. The scattering from isotropically oriented collagen was removed, leaving just the aligned collagen scatter relative to angular position. The data for the aligned collagen were then shifted along the x-axis by 90°, which is consistent with equatorial scattering, yielding a new graph showing preferentially aligned scatter intensity versus the angle of collagen orientation (Figure 2C). The data were also plotted as polar vector graphs (Figure 2D), depicting the preferred orientation of collagen as an average through the tissue thickness at a particular position on the specimen. The radial extent of a polar plot in a particular direction is proportional to the number of fibrils preferentially aligned in that direction. Individual patterns were assimilated to produce two-dimensional maps of collagen arrangement across the whole tissue. In addition, total collagen scatter from each diffraction pattern was found by integrating the total area under the scattering intensity versus rotation angle graph (Figure 2B) and was assembled to produce a contour map representing the amount of fibrillar collagen across the tissue. A contour plot displaying scatter from preferentially aligned collagen was produced in the same way, except that only the preferentially aligned scatter (region above the black line in Figure 2B) was integrated.

## Results

**Figure 3:**
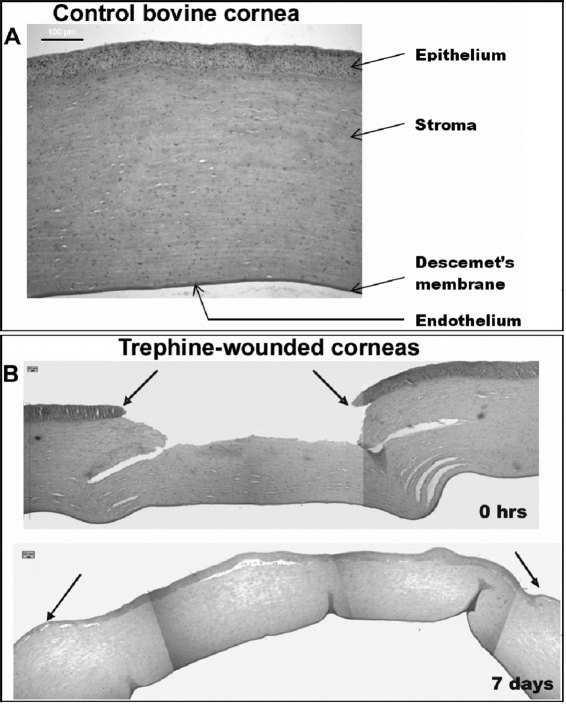
Light microscope images of normal and injured bovine corneas. Cross-sectional images taken from the center of a normal/uninjured bovine cornea (**A**) and a bovine cornea immediately after injury (0 h) and at 1 week post injury (**B**). Black arrows represent the wound edge. Immediately after injury stromal gaps are evident at the wound edge. After 1 week of wound healing the stromal gaps are smaller and epithelial thickening is evident in the wound area. Scale bars for images A and B are 100 μm and 25 μm, respectively.

A light micrograph of a normal bovine cornea at time point 0 h is depicted in Figure 3A. There are only four distinctive layers present since the bovine species does not possess Bowman’s layer. However, this would not be expected to have any implications for epithelial adhesion to the stroma [16]. In wounded samples, some stromal gaps are evident at the wound edge at 0 h (Figure 3B). By the seventh day, complete re-epithelialization is observed. The stromal gap appears smaller, and the epithelium in the wounded area is thicker than in the rest of the cornea, appearing to fill the existing stromal gaps created initially upon wounding.

**Figure 4:**
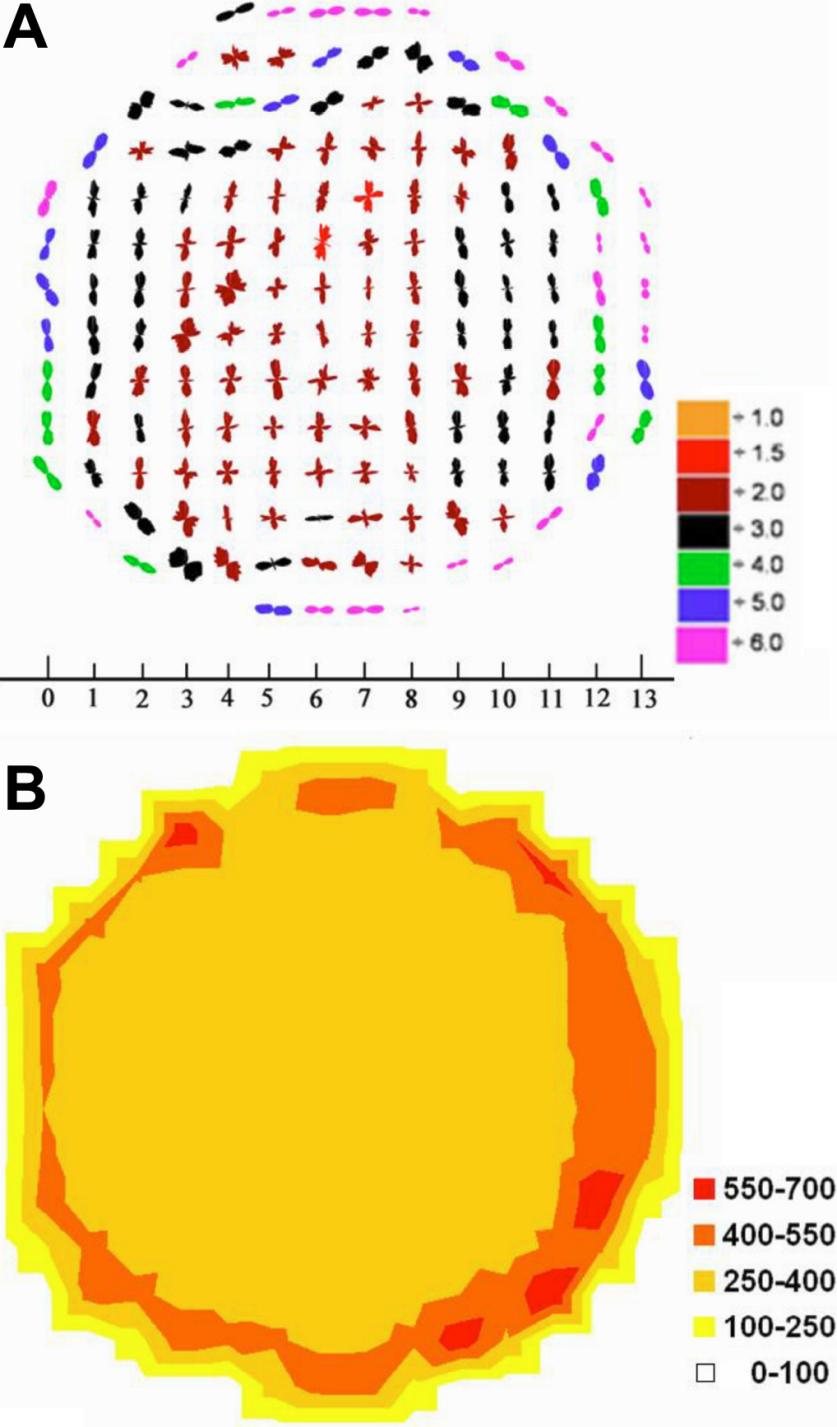
Polar plot map showing the preferred orientation of fibrils in a centrally located 14 mm excised button from an uninjured control bovine cornea. Fibrils tend to have a circular arrangement in the periphery of the tissue and vertical preferred orientation in central areas (**A**). The scale at the bottom is in millimeters. Plots have been scaled down by the factors shown in the color key. The contour map shows the distribution of total fibrillar collagen X-ray scattering in arbitrary units (**B**).

Figure 4A shows the collagen orientation map in an uninjured control bovine cornea. Each one of the polar plots indicates the preferred direction of the collagen fibrils at that specific position on the tissue. Collagen fibrils tend to have a vertical preferred orientation throughout most of the central part of the tissue, indicated by the vertical polar plots. This is in agreement with the results of Hayes et al. [17] who demonstrated a preferential orientation of lamellae in the bovine cornea in the inferior-superior direction. However, in the periphery of the cornea, the plots appear to adopt a more tangential orientation with respect to the edge of the cornea. This effect might have been induced after trimming the tissue to fit the sample holder before scanning. However, it also might be possible that bovine corneas have the same arrangement as in human corneas in that additional fibrils cross the cornea and swamp the contribution from the orthogonal lamellae [5,15]. It can also be seen that when the scaling is taken into account, the polar plots are smaller centrally in the cornea as the tissue is thinner and thus fewer collagen fibrils are in the path of the X-ray beam (Figure 4A). This is also obvious in the contour map (Figure 4B) as total collagen scatter is stronger in the periphery of the cornea. Similar results to those presented in Figure 4 were obtained from the unwounded cornea that had been cultured for one week (data not shown). We have previously shown that this culture system essentially prevents swelling in uninjured corneas [18], and it seems that there are also no changes in collagen arrangement.

**Figure 5:**
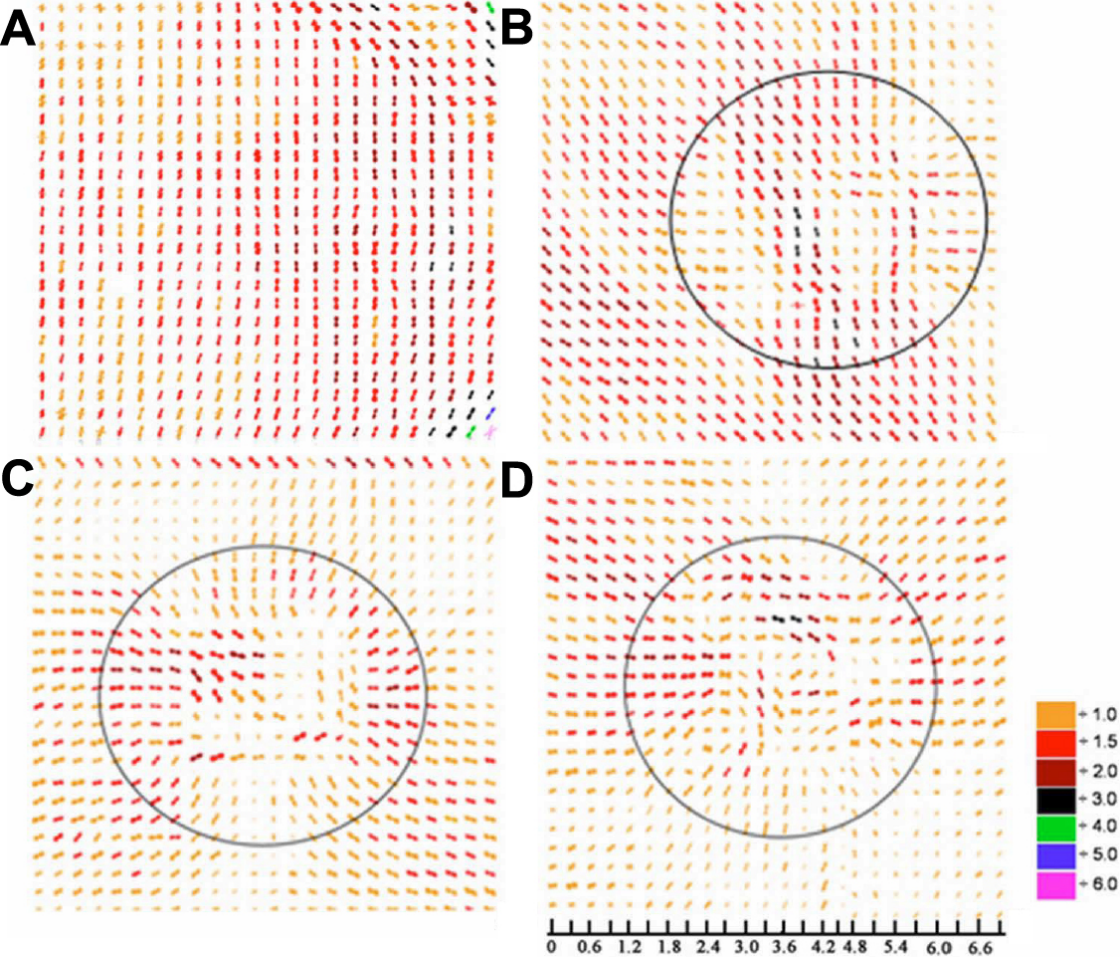
Polar plot maps showing the predominant orientation of collagen in the central region of normal/uninjured and trephine wounded bovine corneas. In the unwounded cornea (**A**), collagen lies predominantly in a superior-inferior direction but immediately after trephine wounding (**B**), a realignment of collagen occurs in the center of the wound. Following 1 (**C**) and 2 (**D**) weeks of wound healing, the initial realignment of collagen post-injury persists within the center of the wound but an additional realignment of collagen occurs both inside and outside the wounded area, where the normal vertical alignment of collagen is replaced by radially aligned collagen. Polar plots have been scaled down by the factors shown in the color key and the solid circle shown in **B**-**D** represents the approximate position of the 5 mm trephine wound. The scale bar is shown in millimeters.

Figure 5 shows high resolution scans from a second uncultured control cornea and three trephine wounded corneas after 0 h, 1 week, and 2 weeks in organ culture. In the high resolution scan of the control/unwounded sample (Figure 5A), the dominant vertical collagen orientation that characterizes the central cornea is evident. A similar result was obtained from three other control specimens (data not shown). Immediately after wounding, the uniaxial orientation of the collagen lamellae is still evident in much of the tissue (Figure 5B), although fibrils are somewhat distorted and seem to adopt a tangential preferred orientation with respect to the edge of the cut near the center of the wound area. This arrangement of collagen fibrils within the wound area is maintained throughout the two-week culture, but after one week, the predominant orientation of collagen around the wound has also altered (Figure 5C,D). Toward the peripheral areas of the cornea, fibrils adopt a radial orientation and lie perpendicular to the tangentially laid fibrils within the wound area, the polar plots also become smaller reflecting a lower degree of collagen alignment in this region (Figure 5C,D).

## Discussion

X-ray diffraction experiments using synchrotron radiation have been widely used to gain important information about the ultrastructure of connective tissue [2,6,13-15,17-21]. Synchrotron radiation is much more intense than common laboratory radiation sources, and therefore, it enables experiments to be performed in a relatively short time. This is important as it facilitates examination of multiple samples and, moreover, minimizes exposure times and hence possible radiation damage or dehydration effects to the samples. X-rays are passed through the entire thickness of the cornea parallel to the optical axis. The X-ray beam has a finite cross-section so X-ray diffraction yields the average values of collagen orientation distributions by averaging the whole depth of the area of the cornea that is examined [11]. By scanning across the tissue, patterns from each position allow spatial variations in collagen orientation to be mapped and to be compared between wounded and unwounded tissues. In this study, we have used wide angle equatorial diffraction, which arises from the lateral packing of the collagen molecules within each fibril. Thus, strictly speaking, the collagen orientation we report is that of the molecules rather than of the fibrils or the lamellae. However, collagen molecules within fibrils are essentially parallel to the fibril axis, and the fibrils themselves run parallel to the lamella direction. Thus, wide-angle scatter gives a very good approximation to the directions of the collagen lamellae in different parts of the tissue.

In the current study, a simple air interface organ culture method was used [10] as this culture system achieves effective re-epithelialization within the first week after injury [10,22]. However, it must be taken into consideration that an in vitro system lacks certain factors essential for the wound healing process. Such parameters are the absence of any tear cytokines, nerve responses, and intraocular pressure fluctuations in response to injury. However, the lack of these parameters has not been an issue in previous in vitro corneal wound healing studies [7,23,24], and, in particular, this has not been related to the orientation of collagen.

The present study has provided important information about the collagen ultrastructural changes in bovine corneas shortly after injury. Although the trephine method of wounding the cornea has previously been used extensively to investigate cellular wound healing processes [23-25], the results presented herein show associated changes in the arrangement of collagen fibrils topically. Polar plot maps revealed a strong tendency of the collagen fibrils in trephine-wounded corneas to form a tangential arrangement with respect to the edge of the cut in a small area within the wound but because this happens from the moment of injury, we believe it is an artifact caused by the need to twist the biopsy punch and subsequently pull upwards on the trephined button to cut it away from the underlying stroma. A similar effect has been noted in the periphery of human trephined buttons [26]. However, the cornea outside the wound is largely unaffected (Figure 5B), and the reorganization observed here at later stages of the culture period is likely to be a result of the healing process and a response of the tissue to wounding. Previous in vivo studies also indicated collagen orientation changes during the healing process within and around the wound in 2 mm full penetrating wounds [6]. Specifically, Connon and Meek [6] showed that in full thickness wounds in rabbits, fibrillar collagen had a strong preferential circularly or tangentially disposed alignment peripheral to and surrounding the wound edge that continued throughout the central wound region.

The polar plots from the trephined wounds were noted to become smaller outside the wound area as the wounds healed (Figure 5C,D). This is unlikely to reflect loss of collagen since no tissue was removed from this area during the culture period. X-ray scattering from collagen can also be influenced by changes in tissue water content, and it is our hypothesis that the smaller plots we have observed here are due to tissue swelling, a phenomenon common during the healing process. It has been noted before in the same culture system that trephine wounded corneas swelled extensively over a two week period in culture whereas uninjured control samples remained normal without any obvious swelling effects [18]. However, previous studies have also shown that even excessive stromal swelling does not affect collagen orientation in the cornea [27], and therefore, the changes in collagen organization observed in the current study were not an outcome of the extensive swelling of the tissue during the healing process.

Figure 5C,D also revealed a radial realignment of collagen in trephine wounded samples with collagen fibrils bending toward the wound area in the middle of the tissue at later stages of the organ culture. It is likely that this specific collagen arrangement is the result of wound contraction that occurs as a normal response to injury during the wound healing process [28-30]. Wound contraction is a cell dependent process and involves the development of muscle-like tension within the wound that pulls together the wound margins [31-33], presumably altering collagen orientation in the cornea during the healing process. This change in collagen organization could also occur as a natural response of the tissue to reinforce the central area of the cornea that becomes considerably thinner upon wounding with the trephine. In general, collagen orientation changes are very common during the healing process in all epithelial tissues, and they have been noted not only in the cornea but in the skin, tendon, and aorta [34-36].

In this study we have shown that remarkable collagen orientation changes occur during the healing process of trephine wounds. It appears that this type of injury plastically deforms the collagen lamellae after wounding, and this would be expected to influence the biomechanical properties of the tissue. Should a similar distortion occur in vivo whenever the cornea is cut and/or manipulated, it is unknown if the tissue would ever recover its original arrangement. Our results suggest it may be worth considering artificially strengthening the anterior cornea before refractive surgery through methods such as cross linking [37,38] in the same way that such methods have been suggested to strengthen the posterior stroma after laser operations on the ocular surface (i.e., LASIK) [39]. A greater knowledge of corneal collagen organization under normal and pathological conditions may also help to facilitate future advances in ocular tissue engineering.
